# Supplementary material for: Nonlinear DNA methylation trajectories in aging male mice
Source: Nat Commun. 2024 Apr 9;15:3074. doi: 10.1038/s41467-024-47316-2 (PMC11004021; doi:10.1038/s41467-024-47316-2)
Supplement: Supplementary file 1 — Supplementary Information [file 41467_2024_47316_MOESM1_ESM.pdf]

Nonlinear DNA methylation trajectories in aging male mice

Supplementary Information

Supp. Fig. 1 – 8

Supp. Table 1

Supp. References

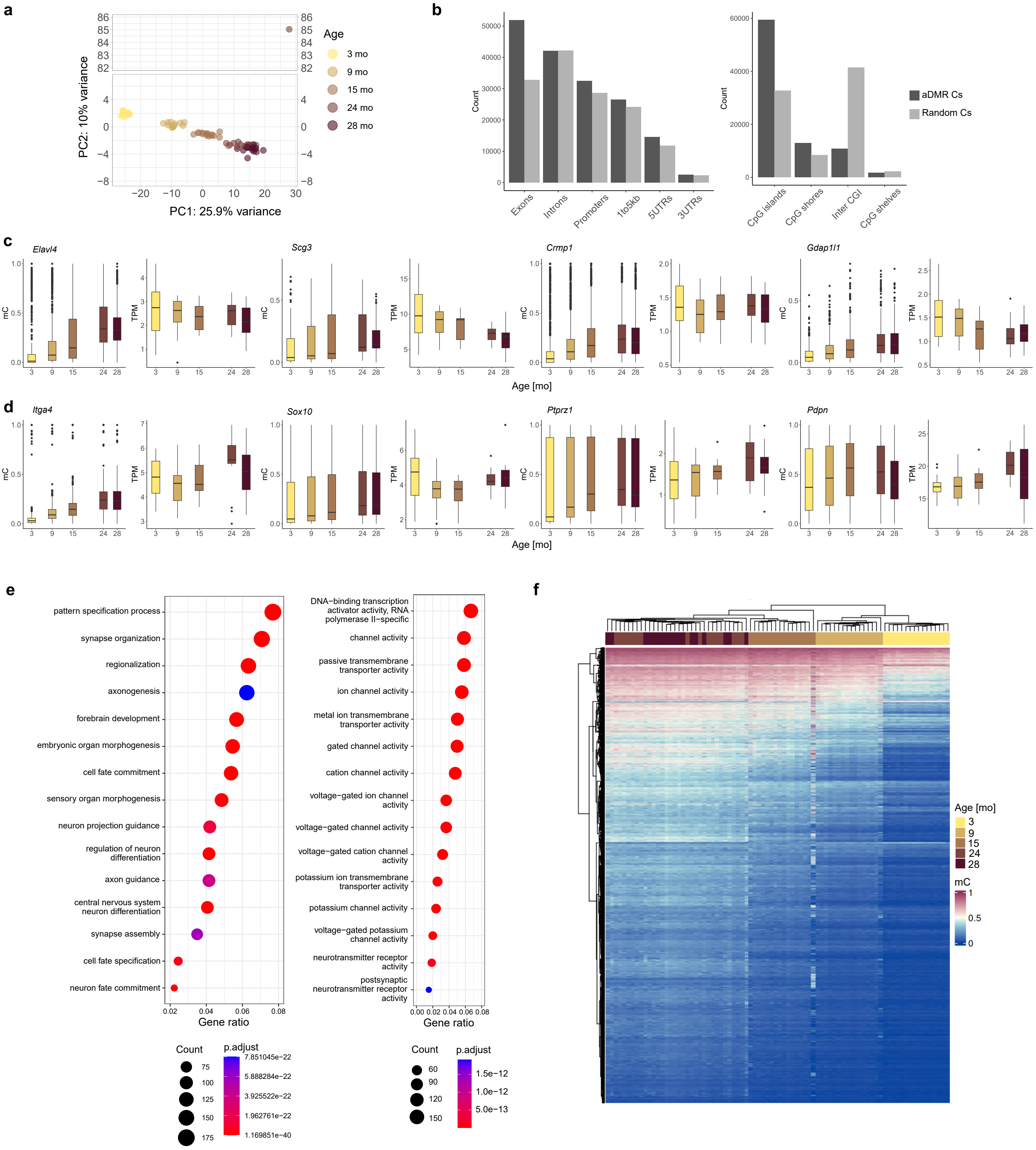

Supplementary Fig. 1 a, Principal component analysis (PCA) based on 10,000 randomly selected CpGs performed with all samples (*n* = 83). **b**, Genic features overlapping with differentially methylated regions in aging (aDMR) CpGs. Promoters - up to 1Kb upstream of the TSS, 1-5kb - 1-5Kb upstream of the TSS. Random CpGs were sampled from all CpGs covered >= 8 replicates. **c**, **d**, Age-related methylation and gene expression changes in markers of enteric neurons (**c**) and glial cells (**d**). For the box plots, the centre line shows the median, the box limits show the first and third quartiles, the upper and lower whiskers extend from the hinge to the largest or the lowest value no further than 1.5× the interquartile range (IQR) from the hinge. *n* = 16 (3 mo, 9 mo, 15 mo, 24 mo), *n* = 18 (28 mo) animals. **e**, Enriched GO categories in biological processes (left) and molecular functions (right) of genes overlapping the aDMRs. FDR-adjusted hypergeometric one-sided P-values are shown. **f**, DNA methylation patterns in 10,000 randomly selected aDMR CpGs with hierarchical clustering of all samples are indicative of three major epigenetic life stages (early-, mid- and late life). Source data are provided as a Source Data file.

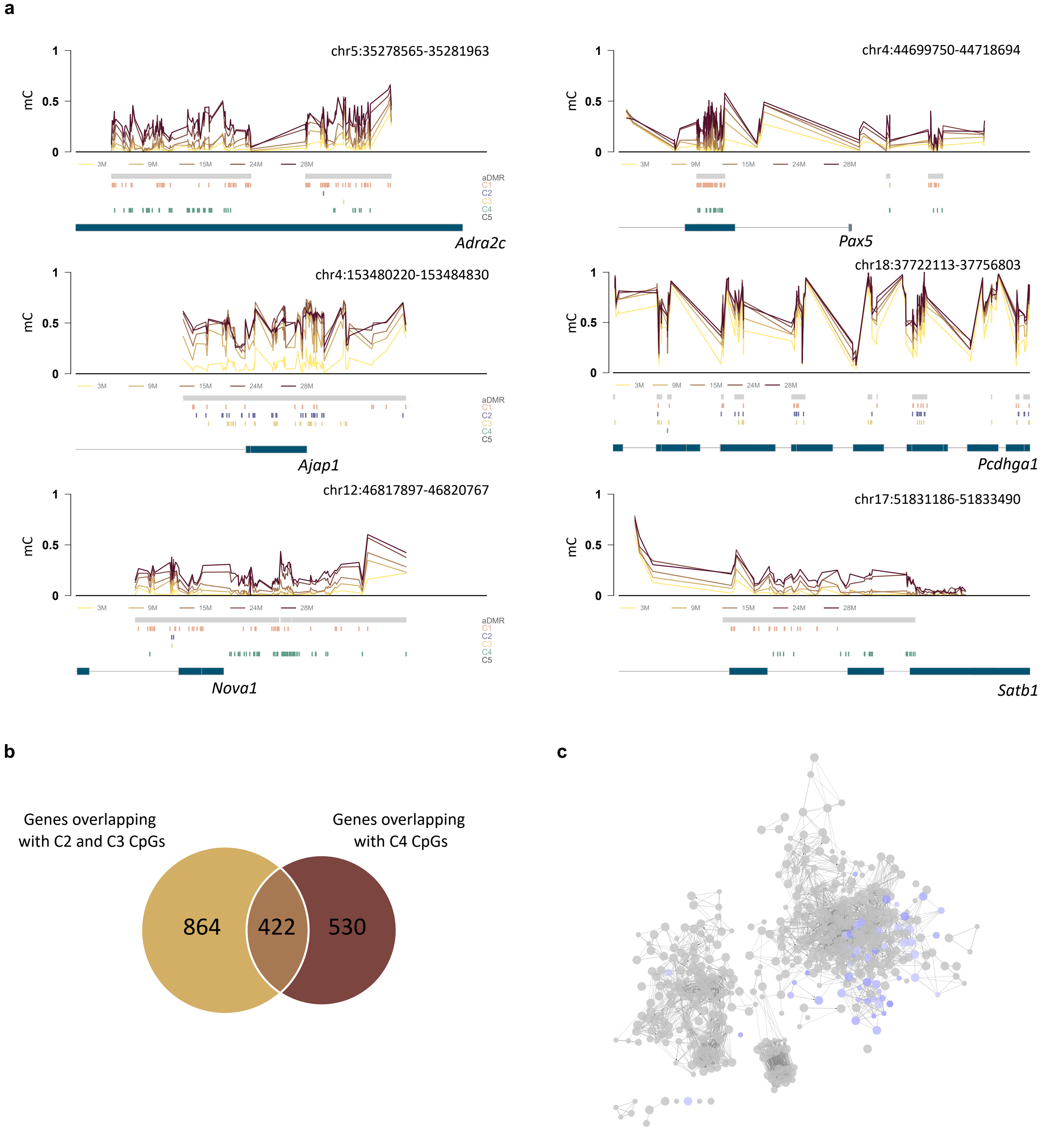

Supplementary Fig. 2 **a**, Methylation patterns in genes associated with early-to-midlife transition (C2 and C3) and mid-to-late-life transition (C4). aDMR - differentially methylated region in aging. **b**, Venn diagram presenting number of genes associated with early-to-midlife clusters C2 and C3 (yellow) and number of genes associated with mid-to-late-life cluster C4 (brown). **c**, Comparative gene ontology analysis of genes associated with clusters C2 and C3 vs C4. Results were functionally grouped and compared. Gray color represents terms common for both transitions, blue represents terms unique for mid-to-late-life transition. No terms were identified as unique for early-to-midlife transition. Source data are provided as a Source Data file.

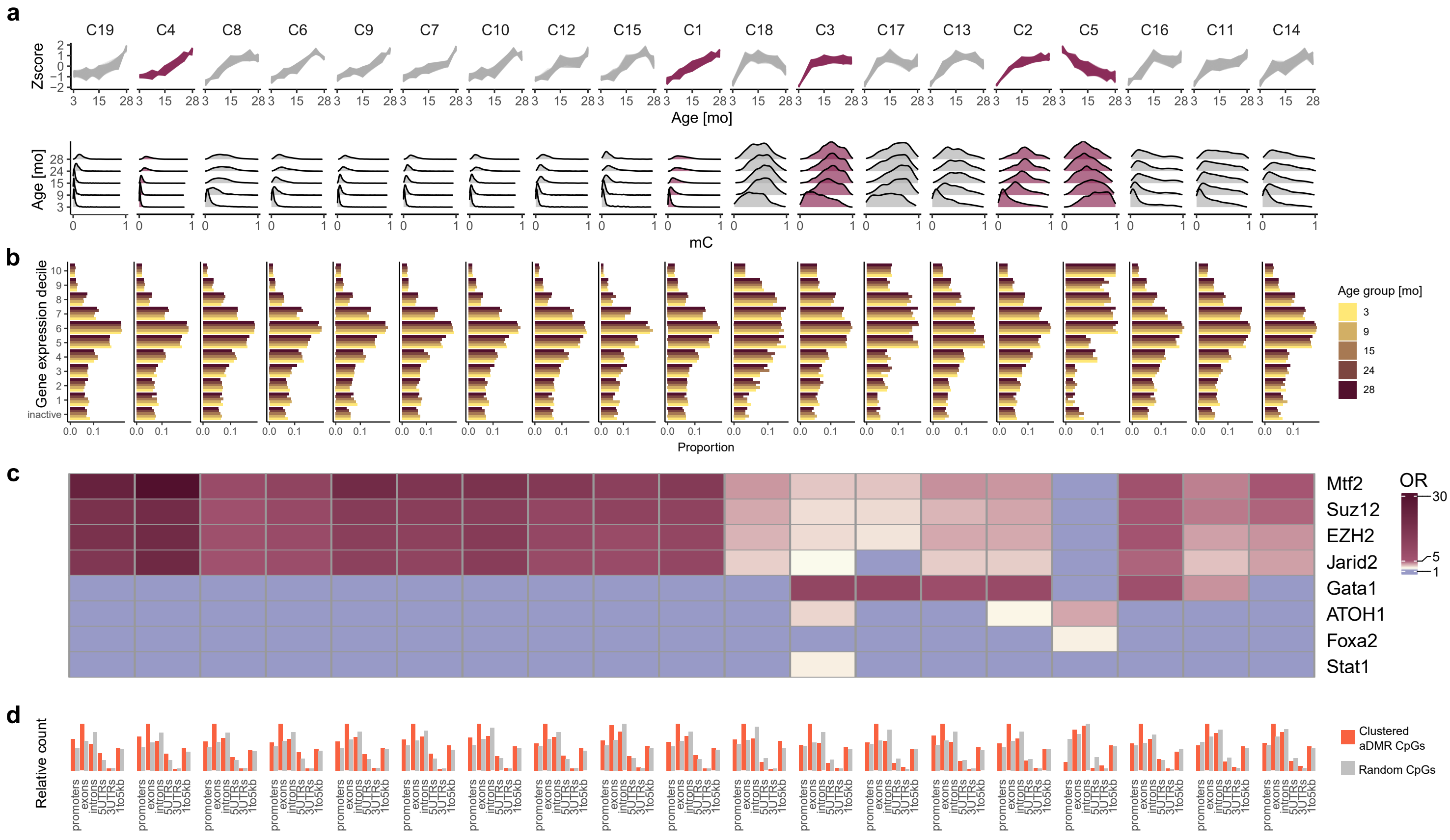

Supplementary Fig. 3 a, DNA methylation trajectories during aging as Z-scores (top) and methylation distribution per age group (bottom). **b**, Gene expression of genes overlapping with aDMR (differentially methylated region in aging) CpGs from different clusters. Proportion of genes in gene expression deciles. Decile limits in TPM for 3, 9, 15, 24, 28 mo mice, respectively: decile 1 - 0.01, 0.01, 0.01, 0.01, 0.01, decile 2 - 0.04, 0.04, 0.04, 0.03, 0.04, decile 3 - 0.1, 0.1, 0.1, 0.1, 0.1, decile 4 - 0.3, 0.3, 0.3, 0.3, 0.3, decile 5 - 1, 0.9, 0.9, 0.9, 0.9, decile 6 - 3.3, 3.2, 3.1, 3.1, 3.2, decile 7 - 9.8, 9.5, 9.4, 9.4, 9.7, decile 8 - 22, 22, 22, 22, 22, decile 9 - 55, 53, 53, 53, 54, decile 10 - 19625, 20394, 18136, 18601, 17720, inactive – mean TPM = 0. **c**, Enrichment of transcription factor binding sites in the methylation clusters. OR - odds ratio. **d**, Genic features overlapping with CpGs from the methylation clusters. Promoter - < 1Kb upstream of the TSS, 1-5kb - 1-5 kb upstream of the TSS. Random CpGs were sampled from all CpGs covered in >= 8 replicates. aDMR - differentially methylated region in aging. Source data are provided as a Source Data file.

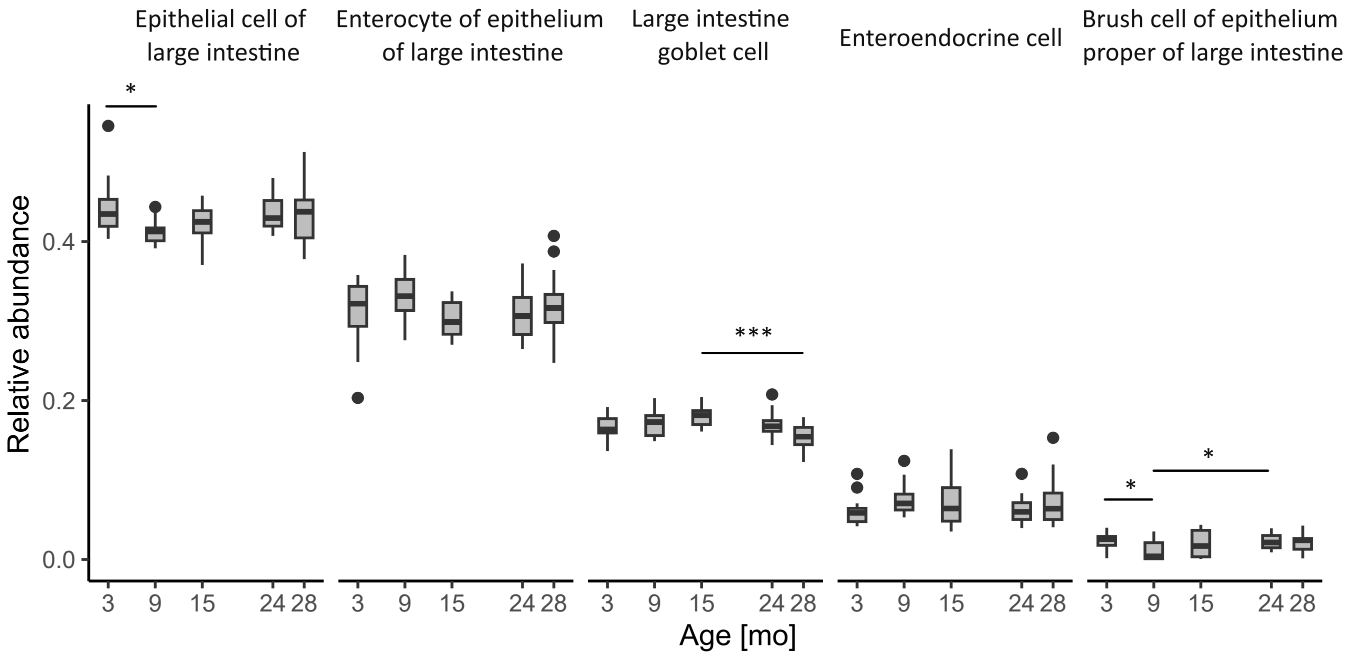

Supplementary Fig. 4 Relative cell type abundance in aging mouse colon inferred from bulk RNA-seq deconvolution analysis. For the box plots, the centre line shows the median, the box limits show the first and third quartiles, the upper and lower whiskers extend from the hinge to the largest or the lowest value no further than 1.5× the interquartile range (IQR) from the hinge. *n* = 16 (3 mo, 9 mo, 15 mo, 24 mo), *n* = 18 (28 mo) animals. *** - adjusted p-value < 0.001, * - adjusted p-value < 0.1. Source data are provided as a Source Data file.

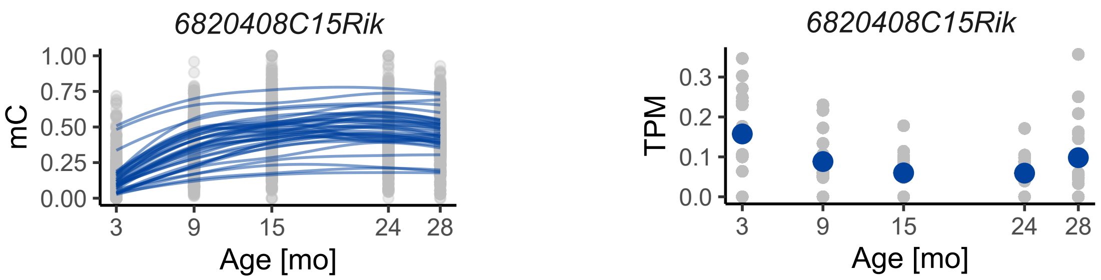

Supplementary Fig. 5 CpGs annotated to *Zcchc3* (Fig. 4c) were also annotated to *6820408C15Rik*. Each smoothed line corresponds to one CpG. Colored dots represent mean TPM for an age group. Source data are provided as a Source Data file.

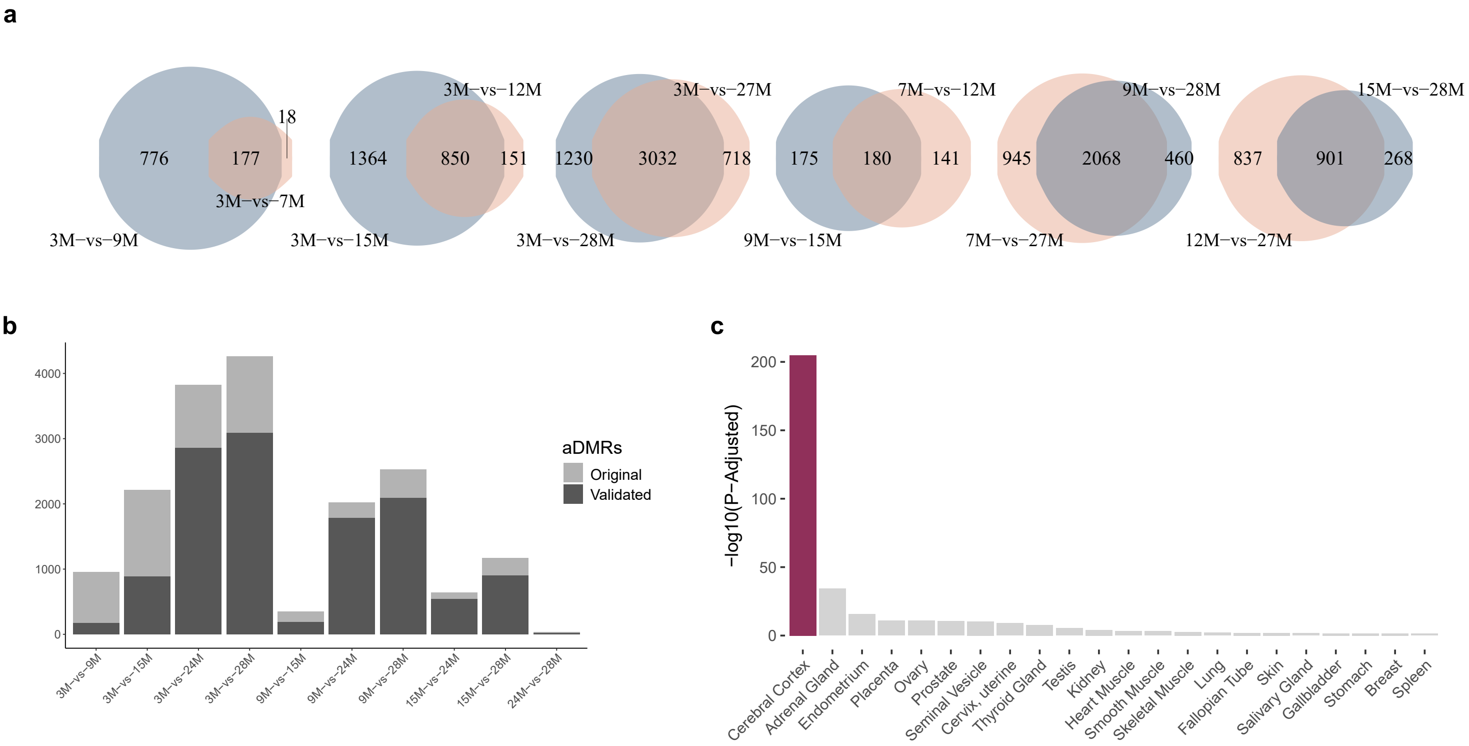

Supplementary Fig. 6 a, Venn diagrams showing the overlap of aDMRs (differentially methylated regions in aging) identified in the original data set (orange) and validation dataset (blue). Shown only for the closest comparisons of age groups, e.g. 3M-vs-9M and 3M-vs-7M. **b**, Number of aDMRs found for each pairwise comparison of age groups (light gray) with number of aDMRs found in the validation data set when comparing the closest age groups (dark gray). **c**, Enrichment of tissue-specific genes in genes associated with the aDMRs found in the validation data set. Source data are provided as a Source Data file.

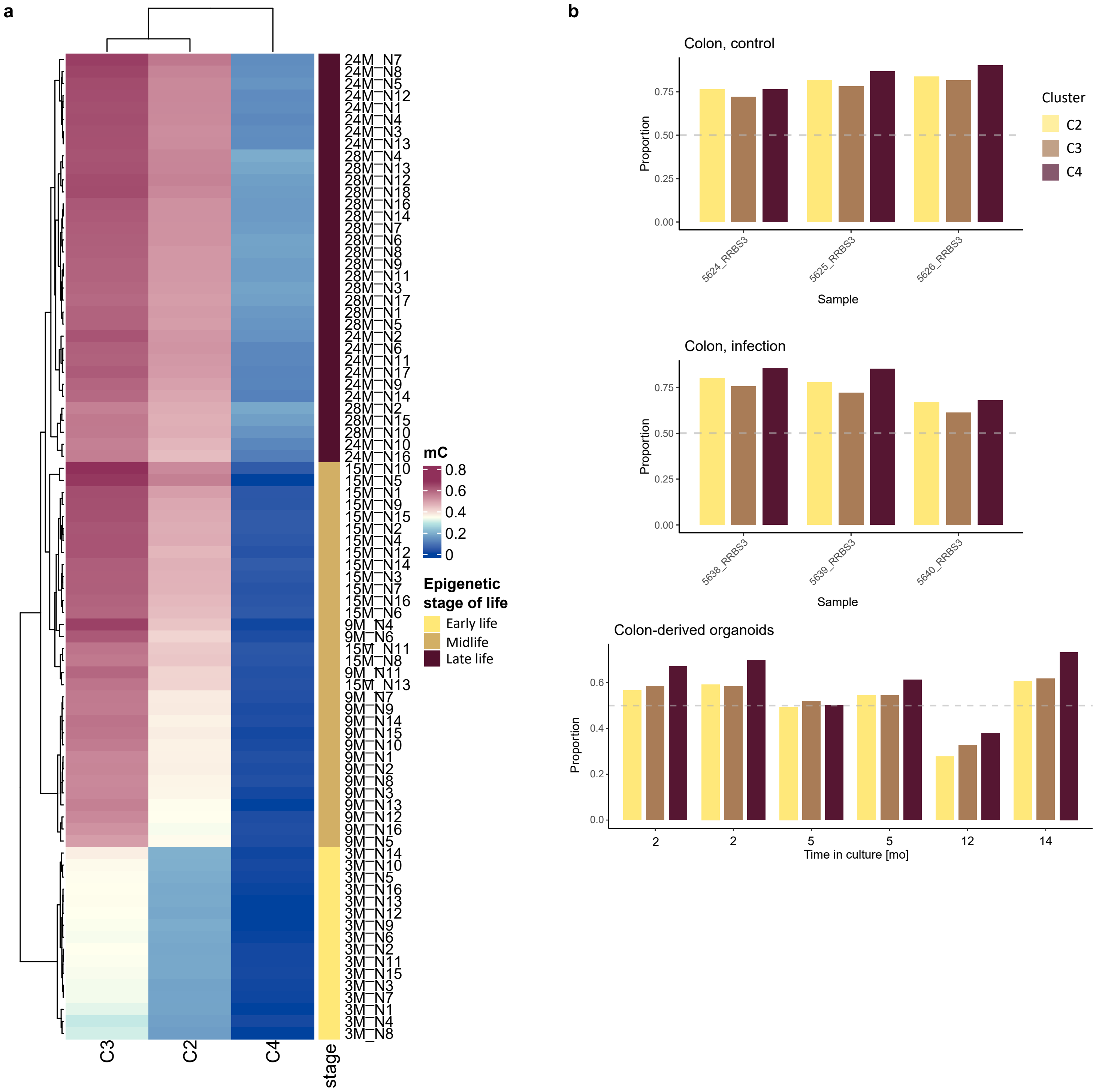

Supplementary Fig. 7 a, Median methylation levels of CpGs from nonlinear clusters C2, C3 and C4 used for training of STageR in the original dataset. **b**, Proportion of CpGs from clusters used by STageR covered in publicly available datasets presented in **Fig. 3** and **Fig. 6**. Source data are provided as a Source Data file.

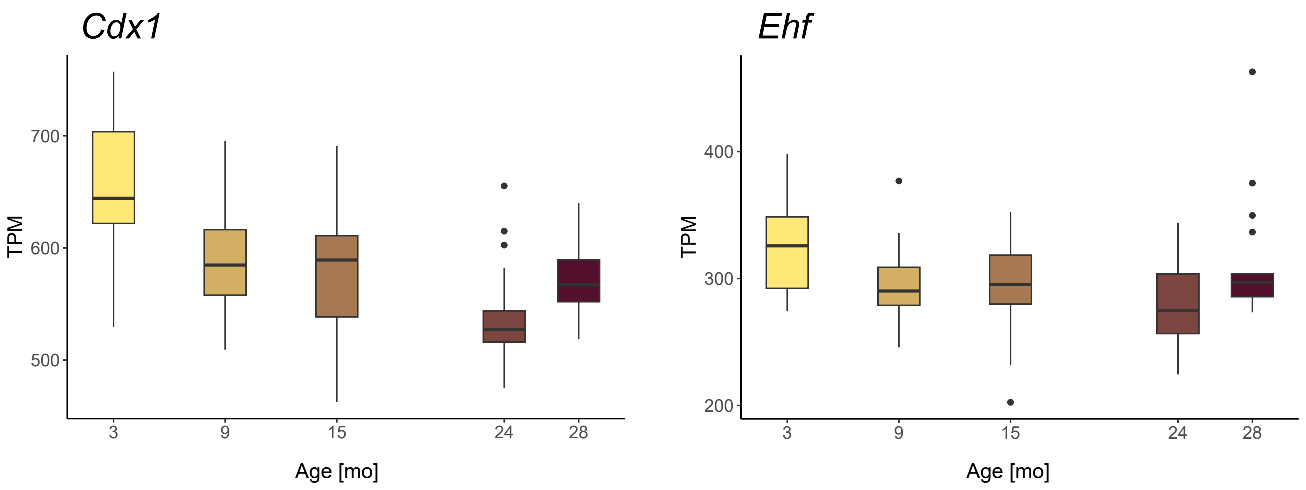

Supplementary Fig. 8 Gene expression of transcription factors Cdx1 and Ehf crucial for colonic differentiation. Decrease in expression during aging is nonlinear and coincides with early-to-midlife (between 3 and 9 months) and mid-to-late-life transition (between 15 and 24 months). For the box plots, the centre line shows the median, the box limits show the first and third quartiles, the upper and lower whiskers extend from the hinge to the largest or the lowest value no further than 1.5× the interquartile range (IQR) from the hinge. *n* = 16 (3 mo, 9 mo, 15 mo, 24 mo), *n* = 18 (28 mo) animals. Source data are provided as a Source Data file.

Supp. Table 1 Genes forming a STRING protein-protein interaction network (Fig. 4d) with known function in intestinal barrier, enteric nervous system and/or colorectal cancer.

	Intestinal barrier	Enteric nervous system	Colorectal cancer
Adamts14			[1]
Ar		[2]	[3]
Cacna1c		[4]	
Ccbe1			[5]
Chat	[6]	[7]	
Cntn1		[8]	
Cyp1b1	[9]		[10]
Efna5			[11]
Ephb1		[12]	[13]
Esm1	[14]		[15]
Eya4			[16]
Fgf12		[17]	[18]
Fgf5		[19]	
Fgfr1			[20]
Flt4			[21]
Foxa2			[22,23]
Foxc1			[24,25]
Foxc2	[26]		
Foxf1	[27]	[28]	[29]
Gata2		[30]	[31]

	Intestinal barrier	Enteric nervous system	Colorectal cancer
Gata4	[32]		[33]
Gdf10		[34]	[35]
Grik2		[17]	
Grik5		[17]	[36]
Hoxc4		[34]	[37]
Itga11			[38]
Lox			[39]
Meis1			[40]
Meis2		[34]	[41,42]
Mn1			[43]
Nefl	[44]	[19]	
Neurod1		[19]	[45]
Nkx2-3			[46]
Ntn1	[47]	[48]	[49,50]
Pde1c		[51]	
Ptgis			[52]
Reln	[53]	[54]	[55]
Sall4			[56]
Sema6d		[57]	
Shank3	[58]	[59]	

Supplementary References

1. Wang, Y. et al. The expression of ADAMTS14 is regulated by promoter DNA methylation and is associated with poor prognosis in colorectal cancer. Exp. Cell Res. 410, 112953 (2022).
2. Rastelli, D. et al. Diminished androgen levels are linked to irritable bowel syndrome and cause bowel dysfunction in mice. J. Clin. Invest. 132, (2022).
3. Xia, T. et al. Androgen receptor gene methylation related to colorectal cancer risk. Endocr Connect 8, 979–987 (2019).
4. Hirst, C. S. et al. Ion channel expression in the developing enteric nervous system. PLoS One 10, e0123436 (2015).
5. Song, J. et al. CCBE1 promotes tumor lymphangiogenesis and is negatively regulated by TGFβ signaling in colorectal cancer. Theranostics 10, 2327–2341 (2020).
6. You, X.-Y. et al. Intestinal Mucosal Barrier Is Regulated by Intestinal Tract Neuro-Immune Interplay. Front. Pharmacol. 12, 659716 (2021).
7. Johnson, C. D. et al. Deletion of choline acetyltransferase in enteric neurons results in postnatal intestinal dysmotility and dysbiosis. FASEB J. 32, 4744–4752 (2018).
8. Veny, M. et al. Contactin-1 Is Required for Peripheral Innervation and Immune Homeostasis Within the Intestinal Mucosa. Front. Immunol. 11, 1268 (2020).
9. Alhouayek, M., Gouveia-Figueira, S., Hammarström, M.-L. & Fowler, C. J. Involvement of CYP1B1 in interferon γ-induced alterations of epithelial barrier integrity. Br. J. Pharmacol. 175, 877–890 (2018).
10. Chen, C. et al. CYP1B1 inhibits ferroptosis and induces anti-PD-1 resistance by degrading ACSL4 in colorectal cancer. Cell Death Dis. 14, 271 (2023).
11. Li, S. et al. MiR-645 promotes invasiveness, metastasis and tumor growth in colorectal cancer by targeting EFNA5. Biomed. Pharmacother. 125, 109889 (2020).
12. Xiong, Y. et al. Eph/ephrin signalling serves a bidirectional role in lipopolysaccharide-induced intestinal injury. Mol. Med. Rep. 18, 2171–2181 (2018).
13. Kim, Y., Ahmed, S. & Miller, W. T. Colorectal cancer-associated mutations impair EphB1 kinase function. J. Biol. Chem. 299, 105115 (2023).
14. Naschberger, E. et al. Analysis of the interferon-γ-induced secretome of intestinal endothelial cells: putative impact on epithelial barrier dysfunction in IBD. Front Cell Dev Biol 11, 1213383 (2023).
15. Kim, J. H. et al. Expression of endothelial cell-specific molecule-1 regulated by hypoxia inducible factor-1α in human colon carcinoma: impact of ESM-1 on prognosis and its correlation with clinicopathological features. Oncol. Rep. 28, 1701–1708 (2012).
16. Kim, S.-J. et al. EYA4 Acts as a New Tumor Suppressor Gene in Colorectal Cancer. Mol. Carcinog. 54, 1748–1757 (2015).
17. Drokhlyansky, E. et al. The Human and Mouse Enteric Nervous System at Single-Cell Resolution. Cell 182, 1606–1622.e23 (2020).
18. Gao, X. et al. Depletion of Fibroblast Growth Factor 12 Restrains the Viability, Stemness, and Motility of Colorectal Cancer. Biomed Res. Int. 2022, 9948461 (2022).
19. Vohra, B. P. S. et al. Differential gene expression and functional analysis implicate novel mechanisms in enteric nervous system precursor migration and neuritogenesis. Dev. Biol. 298, 259–271 (2006).
20. Yin, F. et al. Novel dual inhibitor for targeting PIM1 and FGFR1 kinases inhibits colorectal cancer growth and patient-derived xenografts. Acta Pharm Sin B 12, 4122–4137 (2022).

21. Xiao, X. et al. Genistein suppresses FLT4 and inhibits human colorectal cancer metastasis. Oncotarget 6, 3225–3239 (2015).
22. Zeng, X. et al. Promotes Colorectal Cancer Progression and Targets BCL2-Associated X (BAX) Protein. Iran. J. Public Health 52, 306–314 (2023).
23. Wang, B., Liu, G., Ding, L., Zhao, J. & Lu, Y. FOXA2 promotes the proliferation, migration and invasion, and epithelial mesenchymal transition in colon cancer. Exp. Ther. Med. 16, 133–140 (2018).
24. Li, Q. et al. The FOXC1/FBP1 signaling axis promotes colorectal cancer proliferation by enhancing the Warburg effect. Oncogene 38, 483–496 (2019).
25. Liu, J. et al. Forkhead box C1 promotes colorectal cancer metastasis through transactivating ITGA7 and FGFR4 expression. Oncogene 37, 5477–5491 (2018).
26. González-Loyola, A. et al. FOXC2 controls adult lymphatic endothelial specialization, function, and gut lymphatic barrier preventing multiorgan failure. Sci Adv 7, (2021).
27. Yang, F. et al. Rabeprazole destroyed gastric epithelial barrier function through FOXF1/STAT3-mediated ZO-1 expression. Clin. Exp. Pharmacol. Physiol. 50, 516–526 (2023).
28. Ormestad, M. et al. Foxf1 and Foxf2 control murine gut development by limiting mesenchymal Wnt signaling and promoting extracellular matrix production. Development 133, 833–843 (2006).
29. Wang, S. et al. FOXF1 promotes angiogenesis and accelerates bevacizumab resistance in colorectal cancer by transcriptionally activating VEGFA. Cancer Lett. 439, 78–90 (2018).
30. Kang, Y.-N., Fung, C. & Vanden Berghe, P. Gut innervation and enteric nervous system development: a spatial, temporal and molecular tour de force. Development 148, (2021).
31. Chen, L. et al. Expression and prognostic significance of GATA-binding protein 2 in colorectal cancer. Med. Oncol. 30, 498 (2013).
32. Lepage, D. et al. Gata4 is critical to maintain gut barrier function and mucosal integrity following epithelial injury. Sci. Rep. 6, 36776 (2016).
33. Scheurlen, K. M. et al. The NOTCH4-GATA4-IRG1 axis as a novel target in early-onset colorectal cancer. Cytokine Growth Factor Rev. 67, 25–34 (2022).
34. Memic, F. et al. Transcription and Signaling Regulators in Developing Neuronal Subtypes of Mouse and Human Enteric Nervous System. Gastroenterology 154, 624–636 (2018).
35. Slattery, M. L. et al. Genetic variation in bone morphogenetic protein and colon and rectal cancer. Int. J. Cancer 130, 653–664 (2012).
36. Xie, Z. et al. GRIK5 stimulates colon cancer growth and metastasis through cAMP/PKA/CADM3 signaling. Cell Biol. Int. 47, 1259–1266 (2023).
37. Qi, L. et al. The Effects of Differentially-Expressed Homeobox Family Genes on the Prognosis and HOXC6 on Immune Microenvironment Orchestration in Colorectal Cancer. Front. Immunol. 12, 781221 (2021).
38. Gong, Y.-Z. et al. Diagnostic and prognostic values of integrin α subfamily mRNA expression in colon adenocarcinoma. Oncol. Rep. 42, 923–936 (2019).
39. Liu, Y. et al. Lysyl oxidase: A colorectal cancer biomarker of lung and hepatic metastasis. Thorac Cancer 9, 785–793 (2018).
40. Li, Y. et al. Downregulation of MEIS1 mediated by ELFN1-AS1/EZH2/DNMT3a axis promotes tumorigenesis and oxaliplatin resistance in colorectal cancer. Signal Transduct Target Ther 7, 87 (2022).
41. Wan, Z. et al. MEIS2 promotes cell migration and invasion in colorectal cancer. Oncol. Rep. 42, 213–223 (2019).
42. Wang, X. et al. Hypermethylated and downregulated MEIS2 are involved in stemness properties and oxaliplatin-based

chemotherapy resistance of colorectal cancer. J. Cell. Physiol. 234, 18180–18191 (2019).

43. Ho, Y.-J. et al. High expression of meningioma 1 is correlated with reduced survival rates in colorectal cancer patients. Acta Histochem. 121, 628–637 (2019).
44. Gelmez, E., Jeron, A. & Bruder, D. Negative elongation factor: a key factor in the maintenance of intestinal epithelial barrier integrity. Cellular & molecular immunology vol. 19 453–455 (2022).
45. Li, Z. et al. NeuroD1 promotes tumor cell proliferation and tumorigenesis by directly activating the pentose phosphate pathway in colorectal carcinoma. Oncogene 40, 6736–6747 (2021).
46. Yu, W. et al. Genes regulated by Nkx2-3 in sporadic and inflammatory bowel disease-associated colorectal cancer cell lines. Dig. Dis. Sci. 55, 3171–3180 (2010).
47. Aherne, C. M. et al. Neuronal guidance molecule netrin-1 attenuates inflammatory cell trafficking during acute experimental colitis. Gut 61, 695–705 (2012).
48. Ratcliffe, E. M. et al. Enteric neurons synthesize netrins and are essential for the development of the vagal sensory innervation of the fetal gut. Dev. Neurobiol. 71, 362–373 (2011).
49. Mazelin, L. et al. Netrin-1 controls colorectal tumorigenesis by regulating apoptosis. Nature 431, 80–84 (2004).
50. Paradisi, A. et al. Netrin-1 up-regulation in inflammatory bowel diseases is required for colorectal cancer progression. Proc. Natl. Acad. Sci. U. S. A. 106, 17146–17151 (2009).
51. Dharshika, C. & Gulbransen, B. D. Enteric Neuromics: How High-Throughput 'Omics' Deepens Our Understanding of Enteric Nervous System Genetic Architecture. Cell Mol Gastroenterol Hepatol 15, 487–504 (2023).
52. Frigola, J. et al. Hypermethylation of the prostacyclin synthase (PTGIS) promoter is a frequent event in colorectal cancer and associated with aneuploidy. Oncogene 24, 7320–7326 (2005).
53. Carvajal, A. E. et al. Reelin protects from colon pathology by maintaining the intestinal barrier integrity and repressing tumorigenic genes. Biochim. Biophys. Acta Mol. Basis Dis. 1863, 2126–2134 (2017).
54. Böttner, M. et al. Expression and regulation of reelin and its receptors in the enteric nervous system. Mol. Cell. Neurosci. 61, 23–33 (2014).
55. Serrano-Morales, J. M., Vázquez-Carretero, M. D., Peral, M. J., Ilundáin, A. A. & García-Miranda, P. Reelin-Dab1 signaling system in human colorectal cancer. Mol. Carcinog. 56, 712–721 (2017).
56. Jiang, Y. & Tang, Y. SALL4 advances the proliferation and tumor cell stemness of colon cancer cells through the transcription and regulation of ROBO2. Nucleosides Nucleotides Nucleic Acids 1–15 (2023).
57. Roy-Carson, S. et al. Defining the transcriptomic landscape of the developing enteric nervous system and its cellular environment. BMC Genomics 18, 290 (2017).
58. Wei, S.-C. et al. SHANK3 Regulates Intestinal Barrier Function Through Modulating ZO-1 Expression Through the PKCε-dependent Pathway. Inflamm. Bowel Dis. 23, 1730–1740 (2017).
59. Wang, X. et al. The enteric nervous system deficits in autism spectrum disorder. Front. Neurosci. 17, 1101071 (2023).
60. Pontarollo, G. et al. Commensal bacteria weaken the intestinal barrier by suppressing epithelial neuropilin-1 and Hedgehog signaling. Nat Metab 5, 1174–1187 (2023).
61. Nagy, N. et al. Sonic hedgehog controls enteric nervous system development by patterning the extracellular matrix. Development 143, 264–275 (2016).
62. Wu, C., Zhu, X., Liu, W., Ruan, T. & Tao, K. Hedgehog signaling pathway in colorectal cancer: function, mechanism, and therapy. Onco. Targets. Ther. 10, 3249–3259 (2017).
63. Gao, H. et al. A Prognosis Marker SLC2A3 Correlates With EMT and Immune Signature in Colorectal Cancer. Front. Oncol.

11, 638099 (2021).

64. Goldberg, D. et al. Slit/Robo-mediated chemorepulsion of vagal sensory axons in the fetal gut. Dev. Dyn. 242, 9–15 (2013).
65. Dickinson, R. E. et al. Epigenetic inactivation of SLIT3 and SLIT1 genes in human cancers. Br. J. Cancer 91, 2071–2078 (2004).
66. Wright, C. M. et al. scRNA-Seq Reveals New Enteric Nervous System Roles for GDNF, NRTN, and TBX3. Cell Mol Gastroenterol Hepatol 11, 1548–1592.e1 (2021).
67. Han, Y. et al. Increased expression of TBX2 is a novel independent prognostic biomarker of a worse outcome in colorectal cancer patients after curative surgery and a potential therapeutic target. Med. Oncol. 30, 688 (2013).
68. Bubeck, M., Becker, C. & Patankar, J. V. Guardians of the gut: influence of the enteric nervous system on the intestinal epithelial barrier. Front. Med. 10, 1228938 (2023).
69. Aktar, R. et al. The extracellular matrix glycoprotein tenascin-X regulates peripheral sensory and motor neurones. J. Physiol. 596, 4237–4251 (2018).
70. Zhou, Y. et al. TSHZ3 functions as a tumor suppressor by DNA methylation in colorectal cancer. Clin. Res. Hepatol. Gastroenterol. 45, 101725 (2021).
71. Sylvestre, M., Di Carlo, S. E. & Peduto, L. Stromal regulation of the intestinal barrier. Mucosal Immunol. 16, 221–231 (2023).
72. Galván, J. A. et al. TWIST1 and TWIST2 promoter methylation and protein expression in tumor stroma influence the epithelial-mesenchymal transition-like tumor budding phenotype in colorectal cancer. Oncotarget 6, 874–885 (2015).
73. Koesters, R. et al. WT1 is a tumor-associated antigen in colon cancer that can be recognized by in vitro stimulated cytotoxic T cells. Int. J. Cancer 109, 385–392 (2004).
74. Mariani, F. et al. PLZF expression during colorectal cancer development and in normal colorectal mucosa according to body size, as marker of colorectal cancer risk. ScientificWorldJournal 2013, 630869 (2013).
