# Supplementary material for: Nonlinear DNA methylation trajectories in aging male mice
Source: Nat Commun. 2024 Apr 9;15:3074. doi: 10.1038/s41467-024-47316-2 (PMC11004021; doi:10.1038/s41467-024-47316-2)
Supplement: Supplementary file 3 — Description of Additional Supplementary Files [file 41467_2024_47316_MOESM3_ESM.pdf]

Description of Additional Supplementary Files

File name: Supplementary Data 1
Description: List of aDMRs.

File name: Supplementary Data 2
Description: List of clustered CpGs.
