# Supplementary material for: Nonlinear DNA methylation trajectories in aging male mice
Source: Nat Commun. 2024 Apr 9;15:3074. doi: 10.1038/s41467-024-47316-2 (PMC11004021; doi:10.1038/s41467-024-47316-2)
Supplement: Supplementary file 6 — Reporting Summary [file 41467_2024_47316_MOESM6_ESM.pdf]

nature portfolio

Corresponding author(s): Steve Hoffmann

Last updated by author(s): Jan 16, 2024

Reporting Summary

Nature Portfolio wishes to improve the reproducibility of the work that we publish. This form provides structure for consistency and transparency in reporting. For further information on Nature Portfolio policies, see our Editorial Policies and the Editorial Policy Checklist.

Statistics

For all statistical analyses, confirm that the following items are present in the figure legend, table legend, main text, or Methods section.

n/a	Confirmed	
☐	☒	The exact sample size (*n*) for each experimental group/condition, given as a discrete number and unit of measurement
☐	☒	A statement on whether measurements were taken from distinct samples or whether the same sample was measured repeatedly
☐	☒	The statistical test(s) used AND whether they are one- or two-sided<br>*Only common tests should be described solely by name; describe more complex techniques in the Methods section.*
☒	☐	A description of all covariates tested
☐	☒	A description of any assumptions or corrections, such as tests of normality and adjustment for multiple comparisons
☐	☒	A full description of the statistical parameters including central tendency (e.g. means) or other basic estimates (e.g. regression coefficient) AND variation (e.g. standard deviation) or associated estimates of uncertainty (e.g. confidence intervals)
☐	☒	For null hypothesis testing, the test statistic (e.g. *F*, *t*, *r*) with confidence intervals, effect sizes, degrees of freedom and *P* value noted<br>*Give P values as exact values whenever suitable.*
☒	☐	For Bayesian analysis, information on the choice of priors and Markov chain Monte Carlo settings
☒	☐	For hierarchical and complex designs, identification of the appropriate level for tests and full reporting of outcomes
☒	☐	Estimates of effect sizes (e.g. Cohen's *d*, Pearson's *r*), indicating how they were calculated

Our web collection on statistics for biologists contains articles on many of the points above.

Software and code

Policy information about availability of computer code

Data collection	bcl2fastq v2.20.0.422
Data analysis	Sequencing data processing: FastQC v0.11.9, Cutadapt v2.10, segemehl v0.3.4, Samtools v1.12, BamUtil clipOverlap v1.0.14, haarz v0.3.0, PrinSeq Lite v0.20.4, Hisat2 v2.1.0, UMI-tools v1.1.1<br><br>Data analysis: R 4.1., BEDTools v2.29.2, metilene v0.2.8, clust v1.12.0, annotatr v1.20.0, TxDb.Mmusculus.UCSC.mm10.knownGene v3.10.0, GenomicRanges v1.46.1, ComplexHeatmap v2.10.0, TissueEnrich v1.14.0, clusterProfiler v4.2.2, biomaRt v2.50.3, org.Mm.eg.db v3.14.0, GeneOverlap v1.30.0, Cytoscape v3.8.2, ClueGO v2.5.8, Ingenuity Pathway Analysis v70750971, StringApp v2.0.1, MuSiC v1.0.0, DESeq2 v1.40.2<br><br>STageR: [https://github.com/Hoffmann-Lab/STageR]<br>used by STageR: glmnet v4.1.6, caret v6.0.93, GenomicRanges v1.46.1<br><br>Visualization: ggplot2 v3.4.0, ggridges v0.5.4, Gviz v1.38.4, eulerr v6.1.1., ComplexHeatmap v2.10.0

For manuscripts utilizing custom algorithms or software that are central to the research but not yet described in published literature, software must be made available to editors and reviewers. We strongly encourage code deposition in a community repository (e.g. GitHub). See the Nature Portfolio guidelines for submitting code & software for further information.

Data

Policy information about availability of data

All manuscripts must include a data availability statement. This statement should provide the following information, where applicable:

- Accession codes, unique identifiers, or web links for publicly available datasets
- A description of any restrictions on data availability
- For clinical datasets or third party data, please ensure that the statement adheres to our policy

The RRBS data generated in this study have been deposited in the Gene Expression Omnibus (GEO) database under accession code GSE233734 [https://www.ncbi.xyz/geo/query/acc.cgi?acc=GSE233734]. The RNA-Seq data generated in this study have been deposited in the GEO database under accession code GSE248002 [https://www.ncbi.xyz/geo/query/acc.cgi?acc=GSE248002]. The RRBS data from colon samples upon induced intestinal inflammation used in this study are available in the GEO database under accession code GSE163037 [https://www.ncbi.xyz/geo/query/acc.cgi?acc=GSE163037]. The RRBS data from colon organoids used in this study are available in the GEO database under accession code GSE114801 [https://www.ncbi.xyz/geo/query/acc.cgi?acc=GSE114801]. Mouse reference genome GRCm38 used in this study is available at [https://ftp.ensembl.org/pub/release-102/fasta/mus_musculus/dna/] and [https://ftp.ensembl.org/pub/release-102/gtf/mus_musculus/]. Source data are provided with this paper.

Research involving human participants, their data, or biological material

Policy information about studies with human participants or human data. See also policy information about sex, gender (identity/presentation), and sexual orientation and race, ethnicity and racism.

Reporting on sex and gender	n/a
Reporting on race, ethnicity, or other socially relevant groupings	n/a
Population characteristics	n/a
Recruitment	n/a
Ethics oversight	n/a

Note that full information on the approval of the study protocol must also be provided in the manuscript.

Field-specific reporting

Please select the one below that is the best fit for your research. If you are not sure, read the appropriate sections before making your selection.

☒ Life sciences ☐ Behavioural & social sciences ☐ Ecological, evolutionary & environmental sciences

For a reference copy of the document with all sections, see nature.com/documents/nr-reporting-summary-flat.pdf

Life sciences study design

All studies must disclose on these points even when the disclosure is negative.

Sample size	No statistical method was used to predetermine sample size. Sample size is showed in the figure legends and Methods. Results were statistically significant without showing large variance and did not require larger groups. Samples for the discovery data set were generated in the context of a larger study investigating multiple variables. We estimated the number of samples to be included in this work based on our experience with RRBS data.
Data exclusions	One sample from an original dataset (a 24-month-old mouse) was excluded from downstream analysis as it was identified as an outlier in the principal component analysis (Supplementary Fig. 1a).
Replication	Nonlinear DNA methylation trajcetories as well as STageR were validated with samples from an independet mouse cohort (n=20).
Randomization	Randomization was not applicable to this study. Mice were grouped by age.
Blinding	Blinding was not relevant, because the aim of the study was to compare DNA methylation between different age groups.

Reporting for specific materials, systems and methods

We require information from authors about some types of materials, experimental systems and methods used in many studies. Here, indicate whether each material, system or method listed is relevant to your study. If you are not sure if a list item applies to your research, read the appropriate section before selecting a response.

Materials & experimental systems

n/a	Involved in the study
☒	☐ Antibodies
☒	☐ Eukaryotic cell lines
☒	☐ Palaeontology and archaeology
☐	☒ Animals and other organisms
☒	☐ Clinical data
☒	☐ Dual use research of concern
☒	☐ Plants

Methods

n/a	Involved in the study
☒	☐ ChIP-seq
☒	☐ Flow cytometry
☒	☐ MRI-based neuroimaging

Animals and other research organisms

Policy information about studies involving animals; ARRIVE guidelines recommended for reporting animal research, and Sex and Gender in Research

Laboratory animals	Original RRBS dataset: male C57BL/6J/Ukj mice at ages 3 (n = 16), 9 (n = 16), 15 (n = 16), 24 (n = 17), and 28 (n = 18) months.<br>Validation RRBS dataset: male C57BL6/J mice at ages 3 (n = 4), 7 (n = 5), 12 (n = 5), and 27 (n = 6) months.
Wild animals	The study did not involve wild animals.
Reporting on sex	The analysis was performed on male mice. Inclusion of both sexes was not possible because of limited resources.
Field-collected samples	The study did not involve samples collected from the field.
Ethics oversight	All studies were performed in strict compliance with the recommendations of the European Commission for the protection of animals used for scientific purposes and with the approval of the local government Thüringer Landesamt für Verbraucherschutz, Germany (licenses: TVA 02-024/15, FLI-17-024 and FLI-19-012).

Note that full information on the approval of the study protocol must also be provided in the manuscript.
